# Combining Vocation and Vacation

**DOI:** 10.5812/traumamon.16136

**Published:** 2014-01-25

**Authors:** Shahram Nazerani

**Affiliations:** 1 Firuzgar Medical Center, Iran University of Medical Sciences, Tehran, IR Iran

**Keywords:** Congresses as Topic, Organizations, Iran

**Figure 1.**
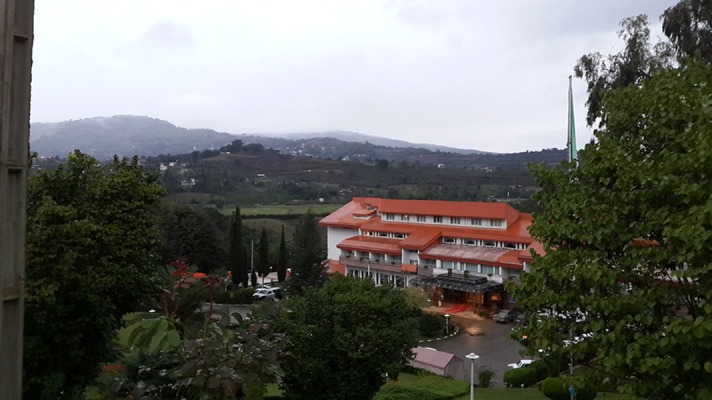
The Hotel Complex in Mazandaran Located in Hirkani Forest in Northern Iran

During my much needed vacation in northern Iran, at the first leg of the journey, we stayed at a hotel complex in Mazandaran province and after a few days traveled to Golestan province and stayed at a hotel in Sari, the capital of the province. The hotel in Mazandaran was located in the forest and had a fabulous, breathtaking view and clean mountain air. The chirping of the birds on the trees was like music to our tired ears still filled with the noise of Tehran (Figure 1). The internet facility was superb, the staff was very polite and hospitable; and the food was excellent. The other hotel in Golestan was also very good. Both hotels had outstanding lecture halls and excellent congress facilities (1). I wondered why we should not use these venues for our Annual Surgical Congresses. Why are our congresses nearly always conducted in Tehran? Why not pursue our vocation in a vacation?

 We can relieve stress, chill-out, relax and enjoy our national countryside with family and friends and participate in our congress as well. The national societies of other countries change the location of their annual congresses every year (2). Although organizing congresses in other cities is not as simple as it is in Tehran, with all its facilities for large venues, shortcomings and facilities of smaller cities will not be recognized until they are encountered. In addition, conducting congresses in other cities has the benefit of disseminating the latest information in our profession to distant provinces more-actively, and provision of more active participation of local medical communities in the congress is provided. During my stay in these two cities, I noted that the quality of life and services in rural areas were lower than those of the capital. The same goes for contemporary treatment modalities we are using in the major cities. As physicians and members of specialty organizations, we have a responsibility to disseminate new information, and diagnostic- therapeutic modalities among our fellow colleagues nationwide; organizing all the congresses or annual gatherings in Tehran does not help the medical communities in distant cities, to become self sufficient in managing congresses. From the tourism point of view, local congresses can boost the economy of that city (3). I believe the scientific societies should organize their meetings in different cities on a rotational basis. The added benefit is that we will travel, relax, meditate and integrate our vocation within a vacation.
